# Production of Prenylated Stilbenoids in Hairy Root Cultures of Peanut (*Arachis hypogaea*) and Its Wild Relatives *A. ipaensis* and *A. duranensis* via an Optimized Elicitation Procedure

**DOI:** 10.3390/molecules25030509

**Published:** 2020-01-24

**Authors:** Lingling Fang, Tianhong Yang, Fabricio Medina-Bolivar

**Affiliations:** 1 Arkansas Biosciences Institute, Arkansas State University, Jonesboro, AR 72401, USA; lfang@astate.edu (L.F.); tyang@biostrategies-lc.com (T.Y.); 2 Molecular Biosciences Graduate Program, Arkansas State University, Jonesboro, AR 72401, USA; 3 Department of Biological Sciences, Arkansas State University, Jonesboro, AR 72401, USA

**Keywords:** prenylated stilbenoid, arachidin, resveratrol, peanut, orthogonal array design, elicitation, hairy root

## Abstract

Prenylated stilbenoids are phenolic compounds produced in a small number of plants such as peanut (*Arachis hypogaea*) to counteract biotic and abiotic stresses. In addition to their role in plant defense, they exhibit biological activities with potential application in human health. Whereas non-prenylated stilbenoids such as resveratrol are commercially available, the availability of prenylated stilbenoids is limited. To this end, hairy root cultures of peanut were developed as an elicitor-controlled bioproduction platform for prenylated stilbenoids. An orthogonal array design approach led to the elucidation of an optimized elicitation procedure consisting of co-treatment of the hairy root cultures with 18 g/L methyl-β-cyclodextrin, 125 µM methyl jasmonate, 3 mM hydrogen peroxide (H_2_O_2_) and medium supplementation with additional 1 mM magnesium chloride. After 168-h of elicitor treatment, the combined yield of the prenylated stilbenoids arachidin-1, arachidin-2, arachidin-3 and arachidin-5 reached approximately 750 mg/L (equivalent to 107 mg/g DW). Moreover, hairy root cultures from the wild *Arachis* species *A. duranensis* and *A. ipaensis* were developed and shown to produce prenylated stilbenoids upon elicitor treatment. These wild *Arachis* hairy root lines may provide a platform to elucidate the biosynthetic origin of prenylated stilbenoids in peanut.

## 1. Introduction

Peanut (*Arachis hypogaea*) an important food and oilseed crop, is believed to have originated from the hybridization between two ancestral wild species, *A. duranensis* and *A. ipaensis.* The production of stilbenoids, including resveratrol and prenylated stilbenoids, as phytoalexins against microbial infection has been reported in peanut [1,2,3]. However, the production of prenylated stilbenoids in wild *Arachis* species has not been studied.

The majority of the stilbenoids in peanut are prenylated. In addition to their defense role in the plant, these stilbenoid derivatives have shown a diverse range of biological activities with potential application to human health. Two major prenylated stilbenoids, arachidin-1 and arachidin-3, isolated from peanut kernels upon slicing and incubation with artificial aeration, displayed antioxidant and anti-inflammatory activities similar to their metabolic precursor, resveratrol [4]. 

**Figure 1:**
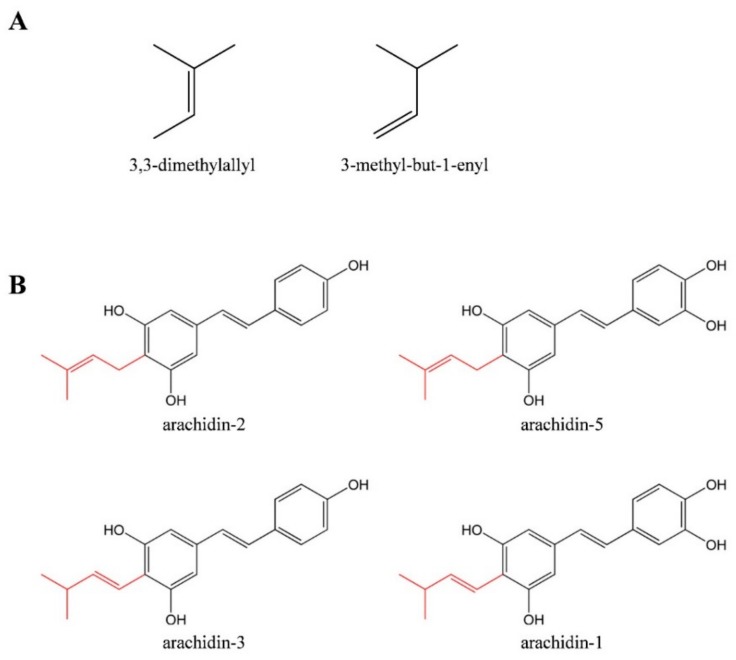
(**A**) Prenylation patterns present on prenylated stilbenoids. (**B**) Chemical structures of four main prenylated stilbenoids identified in elicited peanut hairy root culture. All compounds are shown as their *trans* isomers. Prenylated groups are shown in red.

Recent research also demonstrated the anti-inflammatory activity of arachidin-1 in endothelial cells [5]. The anticancer activity of arachidin-1 isolated from germinated peanut kernels was demonstrated in HL-60 cells through caspase-dependent and caspase-independent pathways [6]. Arachidin-1 and arachidin-3 purified from peanut hairy root cultures exhibited antiviral activity in rotavirus infected HT29.f8 cells by inhibiting rotavirus replication [7]. This antiviral activity was not observed with either of their non-prenylated analogs, piceatannol or resveratrol, suggesting that the antiviral mechanism might depend on the prenylated moiety (3-methyl-1-butenyl moiety) of arachidin-1 and arachidin-3. A cannabinoid receptor binding study of different peanut stilbenoids also demonstrated that the prenylated moiety of arachidin-1 and arachidin-3 improved binding affinity to type II cannabinoid receptor [8]. Despite the diverse bioactivities of arachidin-1 and arachidin-3 demonstrated in in vitro studies, the limited availability of these prenylated stilbenoids has hindered their further progress for in vivo studies. Moreover, the bioactivities of arachidin-5 and arachidin-2, two other prenylated stilbenoids identified from peanut with 3,3-dimethylallyl moiety instead of that on arachidin-1 and arachidin-3 (Figure 1) have not been explored due to their overall low yield.

To increase the availability of prenylated stilbenoids, a peanut cv. Hull hairy root line 3 was developed as a sustainable production platform for inducible stilbenoids and the effect of various elicitors on the production of prenylated stilbenoids was described [9,10]. For instance, co-treatment with methyl jasmonate (MeJA) and methyl-β-cyclodextrin (CD) led a substantial increase in arachidin-1 and arachidin-3 accumulation in the medium of the peanut hairy root culture when compared to either MeJA or CD treatment alone. Recently, peanut hairy root cultures pre-treated with paraquat and then elicited with MeJA and CD were shown to produce resveratrol, arachidin-1 and arachidin-3 [11]. However, elicitation with simultaneous addition of more than two elicitors has not been tested. To optimize the elicitation medium for maximum yield of prenylated stilbenoids, especially arachidin-1 and arachidin-3, we selected the following elicitors: 1) MeJA, a common signaling compound that mediates the induction of the biosynthesis of stilbenoids, 2) CD, an elicitor having capacity of trapping stilbenoids to potentially prevent feedback inhibition and 3) hydrogen peroxide (H_2_O_2_) an inducer of piceatannol accumulation in peanut hairy root cultures [10]. In addition, the concentration of magnesium (Mg^2+^), a co-factor of resveratrol prenyltransferases which are key enzyme involved in prenylated stilbenoid biosynthesis in peanut [12], was also optimized.

Conventionally, one-factor-at-a-time method designs which involve varying one parameter at a time and keeping the others constant are used for optimization of culture medium conditions (i.e., nutrients, temperature, pH, etc.). However, this one-dimensional evaluation usually does not lead to optimal conditions due to ignoring the effects of interaction among the various parameters. On the other hand, full-factorial experimental designs which consider all various parameters and their interactions are extremely time consuming especially for a large number of parameters. Orthogonal arrays are highly fractional factorial experimental designs which separate the individual effects of multiple variables in viable tests with reliable results. These methods have been successfully applied to the optimization of culture media for the enhancement of primary and secondary metabolites production in fermentation processes as reviewed before [13], but not for optimization of elicitor conditions to increase the production of inducible metabolites in hairy root cultures. 

Here, elicitation conditions (i.e., concentrations of H_2_O_2_, Mg^2+^, MeJA, CD and the volume of medium for elicitation) for prenylated stilbenoid production enhancement in peanut hairy root cultures were optimized via multiple steps, including an orthogonal array design method. We also established two hairy root lines from the peanut wild ancestors, *A. duranensis* and *A. ipaensis*, and described for the first time the production of prenylated stilbenoids in these wild *Arachis* species. 

## 2. Results and Discussion

### 2.1. Optimization of Elicitation Conditions for Prenylated Stilbenoid Production in Hairy Root Cultures of Peanut cv. Hull Line 3.

Previous studies have shown that the stilbenoid biosynthetic pathway can be induced by various elicitors in peanut hairy root cultures. Furthermore, most of the stilbenoids secreted to the medium of these cultures [10]. After co-treatment with MeJA and CD for 48~96 h, the average yields of arachidin-1 and arachidin-3 can reach to about 56 and 148 mg/L, respectively [10]. These yields were approximately equivalent to 5.6 and 14.8 mg/g dry weight of peanut hairy root and were much higher than the maximum yield of these compounds isolated from aerated and sliced peanut seeds (0.496 mg/g and 2.415 mg/g dry weight of peanut kernel for arachidin-1 and arachidin-3, respectively) [4]. In the current study, to further increase the levels of prenylated stilbenoids in peanut hairy root, we first evaluated the effect of MeJA, CD, H_2_O_2_ and extra MgCl_2_ in the elicitation medium on the production of arachidin-1 and arachidin-3 using an orthogonal array design method. An L_16_ (4^4^) orthogonal array design with 16 combinations of various concentrations of CD (4.5~27 g/L), MeJA (1~150 µM), H_2_O_2_ (6~120 mM) and extra Mg^2+^ (0~10 mM) was performed in hairy root cultures of peanut cv. Hull line 3. The assignment of parameters with various levels are given in Table S1. The means of arachidin-1 and arachidin-3 yields after 48 h and 72 h treatment of each experimental (Table S2) were used to determine the significant level of each component. In brief, the concentrations of CD and extra MgCl_2_ were optimized to 18 g/L and 1 mM, respectively, while the optimal concentration of MeJA was narrowed down to 100~150 µM. Hydrogen peroxide was found to be the most important determinant factor for arachidin-1 and arachidin-3 production and needed to be tested in lower concentrations. A detailed description of the experimental conditions and orthogonal analysis are described in the Material and Methods section. 

**Figure 2:**
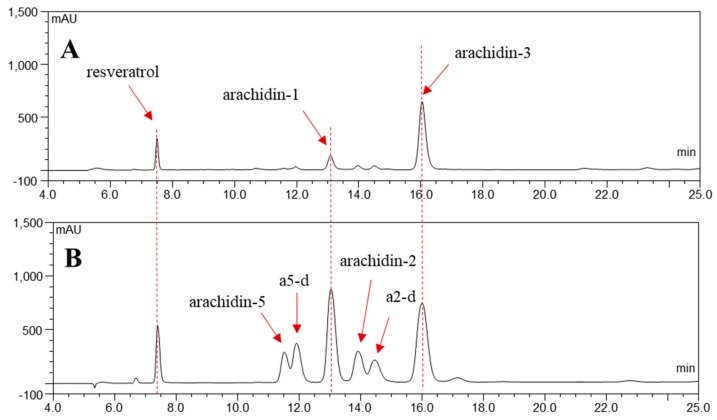
Comparison of secreted stilbenoid profiles before and after elicitation medium optimization. HPLC chromatograms (UV 340 nm) of ethyl acetate extracts from the medium of peanut cv. Hull hairy roots of line 3 after 72 h of treatment with different elicitors: (**A**) 100 µM MeJA 9 g/L CD in a 50 mL elicitation medium and (**B**) 125 µM MeJA, 18 g/L CD, 3 mM H_2_O_2_ and 1 mM MgCl_2_ in a 100 mL elicitation medium. a5-d, arachidin-5 derivative; a2-d, arachidin-2 derivative.

**Figure 3:**
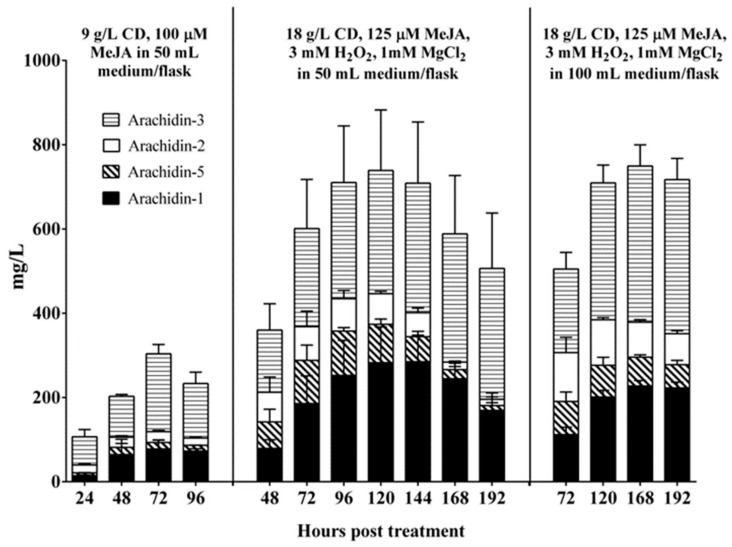
Time course of prenylated stilbenoid yield in the medium of peanut cv. Hull hairy roots of line 3 after treatment with optimized concentration of elicitors. Prenylated stilbenoids were extracted from the culture medium and quantified by HPLC. Yields are expressed in mg/L. Values are the average of three biological replicates and error bars represent standard deviation.

After the first round of optimization using orthogonal array design, we continued to test the combinations of H_2_O_2_ (1.5~6 mM) and MeJA (100~150 µM) with 18 g/L CD and 1 mM MgCl_2_ co-treatment. The yields of arachidin-1 and arachidin-3 after 48 and 72 h of treatment under these conditions are listed in Table S3. The group treated with 3 mM H_2_O_2_ and 125 µM MeJA had the highest yield of arachidin-1 with 231.75 mg/L and the second highest yield of arachidin-3 with 300.76 mg/L, which was slightly lower than the highest yield, 314.30 mg/L in the 1.5 mM H_2_O_2_ and 125 µM MeJA treated group. Thus, the optimal elicitation medium for the production of arachidin-1 and arachidin-3 in peanut hairy root was composed of 18 g/L CD, 125 µM MeJA, 3 mM H_2_O_2_ and additional 1 mM MgCl_2_ in MSV medium. Moreover, despite the original purpose to increase the yields of arachidin-1 and arachidin-3, the yields of arachidin-5, arachidin-2 and other prenylated stilbenoid derivatives (including arachidin-5 derivative and arachidin-2 derivative) were also largely improved in the peanut hairy root cultures upon treatment with this optimal elicitation medium (Figure 2). In order to sustain rapid growth and appropriate gas exchange in a 250 mL Erlenmeyer flask, peanut hairy roots are routinely cultured with 50 mL of liquid medium, and the same volume was used in the elicitation medium. Though, the effect of elicitation medium volume on the overall yields of secreted prenylated stilbenoids in peanut hairy root culture have not been investigated. We observed that the entire root mass of the nine-day-old peanut hairy roots was not completely immersed into the 50 mL elicitation medium resulting in some of the roots to stand above the medium in a 250 mL flask (Figure S1). This might affect the elicitation and secretion of stilbenoids from the roots which were not in contact with the medium and consequently lower the productivity of prenylated stilbenoids from a single flask. Thus, a larger volume (100 mL) of elicitation medium was applied to the peanut hairy roots in a 250 mL flask to ensure that entire root mass was immersed into the culture medium. The yields of arachidin-1 and arachidin-3 were compared with that from 50 mL elicitation medium in a time course experiment. In the 50 mL volume group, the concentrations of arachidin-1 and arachidin-3 reached a plateau around 120 h and started to decline after 144 h (Figure 3). In contrast, arachidin-1 and arachidin-3 from the 100 mL volume group reached their highest concentration after 168 h of treatment with yields of 227.39 ± 12.75 mg/L and 370.59 ± 50.37 mg/L, respectively, and did not show a noticeable decrease at 192 h (Table S4). To evaluate the effect of increasing elicitation medium volumes on the overall production, the yields of prenylated stilbenoids were normalized in mg per gram of root dry weight (mg/g DW) (Figure S2). Under treatment with 100 mL of elicitation medium, the maximum yield of arachidin-1 (32.48 ± 1.82 mg/g DW) and arachidin-3 (52.94 ± 7.20 mg/g DW) were 1.6-fold and 2.4-fold respectively higher than that in the 50 mL volume group. The yields of arachidin-5 (with maximum production of 11.24 ± 3.20 mg/g DW) and arachidin-2 (with maximum production of 16.58 ± 5.17 mg/g DW) from the 100 mL volume group were also much higher than from the 50 mL volume group (Figure S2). After doubling the volume of elicitation medium, the concentration of each prenylated stilbenoid in medium was maintained over time and overall yields of these stilbenoids were about doubled to those in the 50 mL group, indicating that the volume of elicitation medium had more impact on overall yield of secreted prenylated stilbenoids than the biomass of hairy root tissue. 

After these three-step optimization, the final elicitation conditions for the production of arachidin-1 and arachidin-3 were composed of 18 g/L CD, 125 µM MeJA, 3 mM H_2_O_2_ and additional 1 mM MgCl_2_ in 100 mL of MSV medium. The maximum yield of prenylated stilbenoids was approximately 750 mg/L, including 227.39 ± 12.75 mg/L of arachidin-1, 370.59 ± 50.37 mg/L of arachidin-3, 68.38 ± 5.67 mg/L of arachidin-5 and 83.11 ± 6.08 mg/L of arachidin-2, after 168 h treatment. When compared to our previous co-treatment study with CD and MeJA [10], this optimum elicitation conditions provided 5.8- and 4.0-fold increased yields of arachidin-1 and arachidin-3, respectively in a single 250 mL flask. Importantly, the yields of arachidin-5 and arachidin-2 were increased 9.09- and 9.24-fold, thereby providing a high level sustainable bioproduction platform for these 4 types of prenylated stilbenoids. 

### 2.2. Development and Characterization of Arachis ipaensis and A. duranensis Hairy Root Cultures

Cultivated peanut (*Arachis hypogaea* L.) is an allotetraploid derived from two ancestral species, *A. ipaensis* and *A. duranensis.* The recent availability of cultivated peanut and its ancestors’ whole genomes provide a potential for mining candidate genes involved in tolerance/resistance to abiotic and biotic stresses [14,15]. A comparison of the profile of specialized metabolites between the cultivated peanut and its ancestors would provide useful information to elucidate their biosynthesis. Hairy roots of cultivated peanut have been successfully applied not only as a bioproduction platform for prenylated stilbenoids, but for characterization of new genes involved in the biosynthesis of these valuable compounds [16]. Thus, in the present work, hairy root cultures of the wild *Arachis* species *A. duranensis* and *A. ipaensis* were developed and treated with optimized elicitation conditions for prenylated stilbenoid profiling.

To induce hairy roots of *A. ipaensis* and *A. duranensis*, the leaves of 8-week-old seedlings were excised and wounded with *A. rhizogenes*. The wounded inoculated leaves had to be cultured on antibiotic-free MSV medium and subcultured on the same medium to avoid *Agrobacterium* overgrowth until the development of hairy roots, which occurred between 4 to 6 weeks after the inoculation date. Importantly, culture of the inoculated leaves on antibiotic-containing medium prevented hairy root development. 

**Figure 4:**
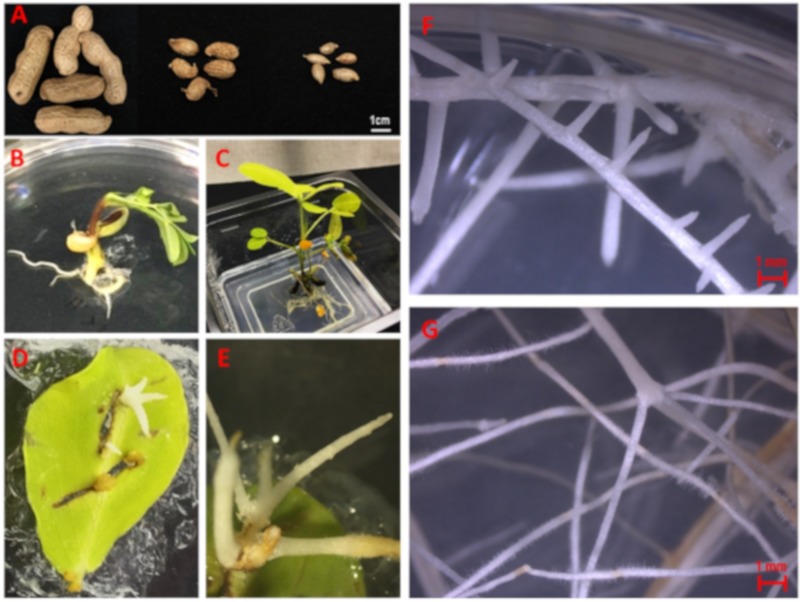
(**A**) Seed pods of *Arachis hypogaea* (left), *A. ipaensis* (center) and *A. duranensis* (right). (**B**) Two-week-old seedling of wild *Arachis*. (**C**) Eight-week-old seedling of wild *Arachis*. (**D**, **E**) Hairy root development from leaf infected with *Agrobacterium rhizogenes*. (**F**) Phenotype of *A. ipaensis* hairy root line E4. (**G**) Phenotype of *A. duranensis* hairy root line B5.

**Figure 5:**
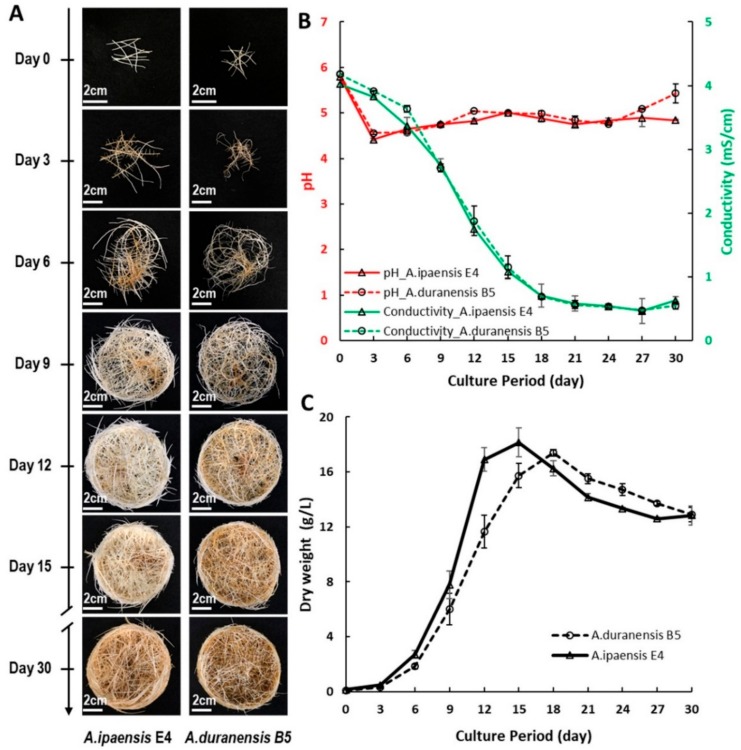
Growth of wild *Arachis* hairy root lines (*A. ipaensis* line AiE4 and *A. duranensis* line AdB5). (**A**) Phenotype of the hairy root during the 30-day culture period. Pictures are representative of triplicate cultures. (**B**) Medium pH (red) and conductivity (green) at different stages of growth. (**C**) Growth curve of the hairy roots. Values represent the average of three independent flasks. Error bars represent standard deviation.

Among several different hairy root lines, *A. ipaensis* E4 (AiE4, Figure 4F) and *A. duranensis* B5 (AdB5, Figure 4G) were selected for further study based on their sustained growth. Furthermore, PCR analysis confirmed the presence of the *rolC* and *aux1* genes, indicating the co-integration of both T-DNAs, T_L_-DNA and T_R_-DNA, of *A. rhizogenes* strain ATCC 15834. The lack of amplification of the *virD2* gene confirmed the absence of any remaining *Agrobacterium* in the root tissue (Figure S3). Growth conditions were monitored for these the newly established wild *Arachis* hairy root lines in MSV medium over a period of 30 days. When compared with line AiE4, the phenotype of the AdB5 line was much curly and thinner in diameter (Figure 5A). Interestingly, the phenotype of *A. ipaensis* line AiE4 was similar to that of the cultivated peanut cv. Hull line 3. 

Figure 5C shows the growth curves of AiE4 and AdB5 hairy root lines. Both lines had a lag phase of about 3 days. A shorter exponential growth phase was observed in line AiE4 (days 3–15) in comparison to AdB5 (days 3–18). The pH and conductivity of the medium were recorded throughout the 30 days of growth to better understand their nutrient utilization. As shown in Figure 5B, the conductivity of the medium decreased while the biomass increased. This suggests that hairy roots were taken up the nutrients from culture medium for their growth. After the hairy roots reached their highest biomass, no changes in conductivity were observed. A similar pattern of pH values was observed for both lines.

### 2.3. Production of Prenylated Stilbenoids in Hairy Root Cultures of Arachis ipaensis and A. duranensis

**Figure 6:**
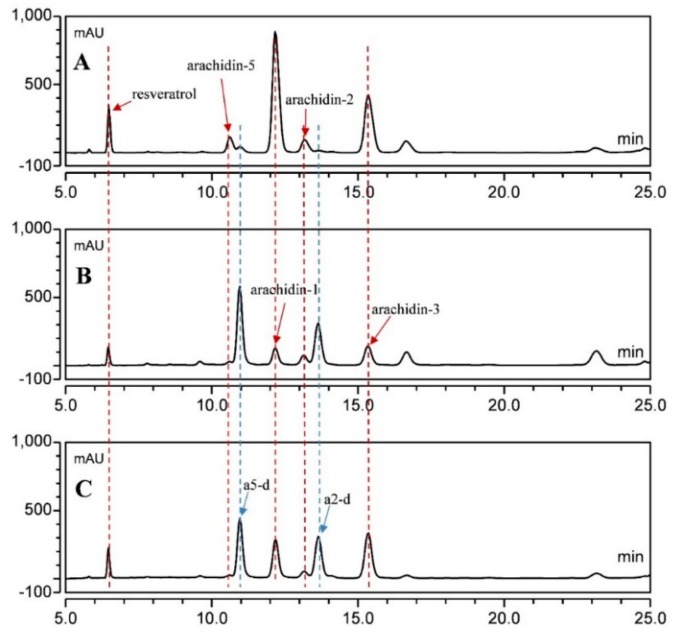
Comparison of secreted stilbenoid profiles between wild Arachis and cultivated peanut hairy root cultures. HPLC chromatograms of extracts from the medium of hairy root cultures of (**A**) peanut cv. Hull line 3, (**B**) *A. duranensis* line AdB5 and (**C**) *A. ipaensis* line AiE4 after 192 h of elicitation treatment. The extracts of lines AdB5 and AiE4 were concentrated 2-fold before HPLC analysis. All chromatograms were monitored at 340 nm.

To determine if the hairy root cultures of the wild *Arachis* species had the ability to produce prenylated stilbenoids, nine-day-old cultures of *A. ipaensis* line AiE4 and *A. duranensis* line AdB5 were treated with the optimal elicitation medium described above. Extracts from the culture medium were prepared after different periods of treatment and analyzed by HPLC (Figure 6). Similarly to the cultivated peanut hairy root line 3, the AiE4 and AdB5 lines were able to produce and secrete resveratrol and various prenylated stilbenoids, including arachidin-5, arachidin-5 derivative, arachidin-1, arachidin-2, arachidin-2 derivative and arachidin-3 into medium upon elicitor treatment. 

**Figure 7:**
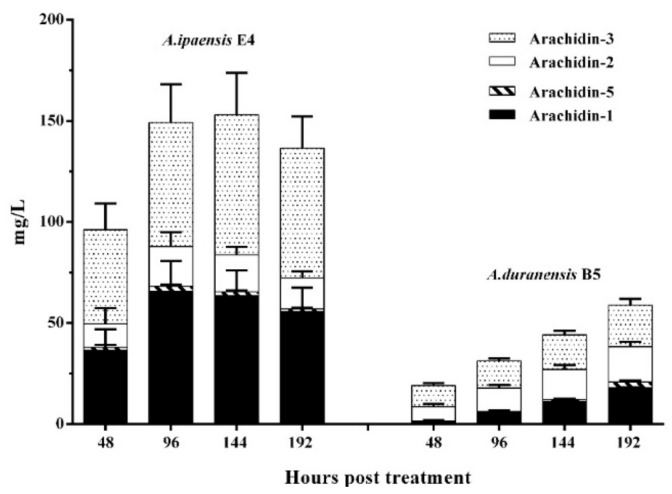
Yield of prenylated stilbenoids in hairy root cultures of *Arachis ipaensis* and *A. duranensis*. Hairy root cultures were elicited 125 µM MeJA, 18 g/L CD, 3 mM H_2_O_2_ and 1 mM MgCl_2_ in a 100 mL elicitation medium. Yield expressed in mg/L and each bar represents the average of 3 biological replicates and error bars represent standard deviation.

The combined yields of arachidin-1, -2, -3, and -5 were approximately 4.0- and 10-fold higher in the cultivated peanut line 3 than in AiE4 and AdB5 lines, respectively (Figure 7, Table S6). The yield of arachidin-1 in peanut line 3 after a 192-h elicitation treatment reached 300.35 ± 36.40 mg/L, which was 5.4- and 16.7-fold higher than that in AiE4 and AdB5 lines, respectively. Whereas the yield of arachidin-3 in line 3 reached 178.04 ± 31.15 mg/L, which was 2.8- and 8.9-fold higher than that in AiE4 and AdB5 lines, respectively (Figure 7, Table S6). Though the levels of arachidin-1, arachidin-2, arachidin-3 and arachidin-5 were higher in the cultivated peanut line 3, the wild *Arachis* lines AiE4 and AdB5 lines secreted a much higher proportion of arachidin-5 derivative and arachidin-2 derivative than the cultivated peanut line 3 (Figure 6). Particularly in the AdB5 line, arachidin-5 derivative and arachidin-2 derivative were the predominant prenylated stilbenoids. Importantly, arachidin-2 derivative and arachidin-5 derivative have been reported as direct enzymatic derivatives of arachidin-2 and arachidin-5 in peanut hairy roots and may play an important role in the biosynthesis of prenylated stilbenoids [12]. The chemical structures of arachidin-5 derivative and arachidin-2 derivative have not been elucidated. Thus, availability of these hairy root lines might provide a platform to produce these arachidin derivatives for further purification and analysis. To our knowledge, this is the first study to show prenylated stilbenoid production in *A. ipaensis* and *A. duranensis*, the two-wild ancestor species of the cultivated peanut *A. hypogaea*, and these hairy root lines offer opportunities to study the evolution of the biosynthesis of this important class of specialized metabolites in peanut. 

## 3. Materials and Methods 

### 3.1. Growth Conditions and Elicitation of Peanut Hairy Root Culture Line 3 

Hairy root line 3 used in this study was previously established from peanut cv. Hull [9] and maintained in 250 mL flasks with 50 mL of MSV media as previously described. Nine-day-old peanut hairy roots were used for yield optimization of prenylated stilbenoids in the culture medium. Prior to elicitation, the spent medium was removed and replaced with 50 mL or 100 mL of fresh MSV medium containing 3% sucrose with different concentration of elicitors as described below. All elicitations were carried out at 28 °C under continuous darkness. For the time course experiment, aliquots of medium from multiple time points were collected from the same flask of elicited hairy root culture.

### 3.2. Orthogonal Array Design for the Optimization of Elicitation Medium

There are four key elicitation factors, MeJA, CD, H_2_O_2_ and MgCl_2_ which may affect the production of prenylated stilbenoids in peanut hairy root cultures. These four factors, each at four levels for the orthogonal array design are listed in Table S1. The level 2 value of CD (9 g/L) was based on the previous study [10]. The level 1, level 3 and level 4 values of CD were half, twice and triple of the level 2 value, respectively. The level values of MeJA and H_2_O_2_ were determined by the preliminary trials (data not shown). The maximum concentration of Mg^2+^ (10 mM of MgCl_2_) used in the orthogonal array was based on the saturated concentration of Mg^2+^ in a previously described flavonoid-specific prenyltransferase reaction [17,18,19,20,21].

The orthogonal array is expressed by its number of rows and columns, as well as by the number of levels in each column. For example, the matrix of an L_16_ (4^4^) array used in this study has 16 rows and 4 four-level columns. Each row represents a special combination with the level of elicitors in the elicitation medium and each column represents various levels of one elicitor. To be orthogonal, each level of elicitor occurs equally often in every column and each level of one elicitor pairs equally with all four levels of other elicitors without pairing repetition (Table S2). 

Based on the orthogonal array, the peanut hairy root culture was elicited with each combination of elicitors. Each elicitor treatment included two biological replicates and 1 mL of culture medium was collected at the 48 h and 72 h time points of elicitation to determine the concentration of stilbenoids. The prenylated stilbenoids were extracted by an equal volume of ethyl acetate and quantified by HPLC as described below. The production of arachidin-1 and arachidin-3 from each combination was recorded on the right side of the orthogonal array in Table S2. 

To determine the optimal concentration of each elicitor for prenylated stilbenoid production, the average values of arachidin-1 and arachidin-3 yields in mg/L for each factor at level *n* (*n* = 1, 2, 3 or 4) after *m* hours (*m* = 48 or 72) treatment were calculated as *k_n_^m^* and k*_n_^m^, respectively (recorded below the orthogonal array in Table S2). In the orthogonal analysis, optimum concentrations of each elicitor for arachidin-1 or arachidin-3 production are those that may result in the largest *k* value. For example, the *k* values of arachidin-1 in the 72 h treatment group for various concentrations of CD, *k_1_^72^*, *k_2_^72^*, *k_3_^72^* and *k_4_^72^* were 85.91, 83.74, 114.07 and 97.07, respectively. As the highest *k* value among others, *k_3_^72^* indicated the optimal concentration of CD for maximizing arachidin-1 production. Therefore, the optimal concentration should be at level 3 (18 g/L). In order to determine the contribution of each elicitor to prenylated stilbenoid production, the absolute difference among *k_n_^m^* and k*_n_^m^ for each factor was measured by *r^m^* and r*^m^, respectively. If the *r* value of one elicitor is higher than the r value of the other elicitors, the concentration of this elicitor in the elicitation medium may have a higher effect on prenylated stilbenoid production when compared to the other elicitors.

For analysis results, in brief, according to the *r* values, the influence of the elicitation medium on the production of arachidin-1 after 48 and 72 h of treatment was H_2_O_2_ >> MeJA > CD ~ MgCl_2_ and H_2_O_2_ > CD > MgCl_2_ > MeJA, respectively. The influence on arachidin-3 production was H_2_O_2_ > MgCl_2_ > CD > MeJA at both 48- and 72-h treatment groups. Hydrogen peroxide was found to be the most important determinant factor for arachidin-1 and arachidin-3 production. The largest values among the *k_n_^m^* and k*_n_^m^ of the H_2_O_2_ factor were *k_1_^48^*, *k_1_^72^*, k*_1_^48^ and k*_1_^72^ indicating that the level 1 value, 6 mM H_2_O_2_, led to the highest yield of arachidin-1 and arachidin-3 when comparing to other levels. These results suggested the potential of oxidative damage from high level of H_2_O_2_ to the peanut hairy root tissue and also highlighted the need to test lower concentrations of H_2_O_2_. Considering as priority the yield of arachidin-1 and arachidin-3 at the 72-h treatment group, the concentration of CD and MgCl_2_ that were additionally added in the elicitation medium were optimized to 18 g/L (level 3) and 1 mM (level 2) respectively due to the highest values of *k^72^* CD (*k_3_^72^* and k*_3_^72^) and MgCl_2_ (*k_2_^72^* and k*_2_^72^). For the MeJA factor, the *k_3_^48^* > *k_4_^48^*, *k_3_^72^* < *k_4_^72^* and k*_3_^48^ > k*_4_^48^, k*_3_^72^ < k*_4_^72^ suggested that the concentration of MeJA needed to be tested further between the 100 µM (level 3) and 150 µM (level 4). 

### 3.3. Seed Sterilization and Germination of Arachis ipaensis and A. duranensis

Seeds of wild peanut *Arachis ipaensis* (accession No. PI 468322, plant name GKBSPSc 30076, country of origin Bolivia) and *Arachis duranensis* (accession No. PI 468372, plant name ScBo 15101, country of origin Paraguay) were obtained from the Germplasm Resources Information Network (GRIN) of the United States Department of Agriculture. After removing the shell, the seeds were soaked in 0.1% Palmolive detergent for 2 min, then followed by 50% commercial bleach solution for 15 min, and rinsed thoroughly with sterilized distilled water for 4–5 times. The sterilized seeds were put on MS (Murashige and Skoog) basal media with 30 g/L sucrose and 4 g/L phytagel and cultured at 24 °C under darkness until germinated.

### 3.4. Establishment of Hairy Root Lines of Arachis ipaensis and A. duranensis 

Leaves were excised from in vitro seedlings and wounded with a scalpel containing *Agrobacterium rhizogenes* strain ATCC 15834. The wounded leaves were cultured on MSV medium [9] and subcultured on fresh MSV medium about every three days (once *Agrobacterium* growth was observed on the plate). The leaves were maintained in this medium until hairy roots developed. Among the several hairy root lines established, line E4 of *Arachis ipaensis* and line B5 from *A. duranensis* were selected for their vigorous and sustained growth and used in further analysis. Genomic DNA was extracted from these hairy roots using the DNeasy^®^ Plant Mini kit (Qiagen, Germantown, MD, USA). PCR analyses of *rolC*, *aux1* and *virD2* genes were performed as described before [22]. To establish a growth curve of the hairy root lines *A. ipaensis* E4 and *A. duranensis* B5, ten 2–3 cm long tips were excised and cultured into 250 mL flasks containing 50 mL of MSV medium. The flasks were placed on an orbital shaker (Innova^®^ 44R, New Brunswick Scientific, Hauppauge, NY, USA) at 90 rpm, 28 °C under continuous darkness. Three flasks of each line were harvested at days 3, 6, 9, 12, 15, 18, 21 24, 27, and 30. The hairy roots were rinsed with tap water and lyophilized for 48 h (Freeze Dry System Freezone 4.5, Labconco™, Kansas City, MO, USA) to obtain the dry weight (DW). The pH and conductivity were recorded from the culture media at each of the time points.

### 3.5. Elicitation of Arachis ipaensis and A. duranensis Hairy Root Cultures 

Nine-day-old *A. ipaensis* hairy root culture line AiE4 and *A. duranensis* hairy root culture line ApB5were used for elicitation. Prior to elicitation, the spent medium was removed and replaced with 100 mL of the optimized elicitation medium described above (MSV medium containing 3% sucrose with 18 g/L CD, 125 µM MeJA, 3 mM H_2_O_2_ and additional 1 mM MgCl_2_). All elicitations were carried out at 28 °C under continuous darkness. Nine-day-old hairy root line of peanut cv. Hull line 3 was elicited under the same conditions as a control group for prenylated stilbenoid production.

### 3.6. Extraction and Analyses of Stilbenoids from the Culture Medium 

A 900 µL aliquot from the elicited culture medium was mixed with 900 µL of ethyl acetate in a 2 mL microcentrifuge tube by vortexing for 30 s. Then 500 µL of the upper organic phase was removed, transferred to an amber HPLC vial and dried under nitrogen stream using a Reacti-Vap III apparatus (Thermo Fisher Scientific, Waltham, MA, USA). The extract was redissolved in either 500 µL or 1 mL of MeOH and analyzed by reverse phase HPLC as detailed below.

HPLC analyses were performed similarly as described before [10]. Briefly, chromatography was done in a SunFire C18, 5 μm, 4.6 × 250 mm column (Waters, Milford, MA, USA) at 40 °C with a flow rate at 1.0 mL/min. The mobile phase consisted of 0.5% formic acid in water (A) and MeOH (B). The column was initially equilibrated with 100% A for 1 min. Then a linear gradient was performed from 60% A and 40% B to 65% A and 35% B (1−20 min), followed by a linear gradient from 65% A and 35% B to 100% B and an isocratic elution in 5 min (20−25 min).

Reference compounds included commercially available *trans*-resveratrol (Biophysica, La Jolla, CA, USA). Arachidin-5, arachidin-1, arachidin-2 and arachidin-3 standards (>95% purify determined by HPLC under absorbance at 320 nm and 340 nm) were purified from elicited peanut hairy root medium as described before [7]. Dilutions of the standards were made in MeOH to obtain calibration curves for quantitative analysis. Calibration curves were established using absorbance at 320 nm for resveratrol and arachidin-2 and arachidin-5, and 340 nm for arachidin-1 and arachidin-3.

## 4. Conclusions

In conclusion, elicitation conditions were optimized to enhance the production of prenylated stilbenoids in peanut hairy roots for the first time via an orthogonal array design approach with multiple elicitors. The optimized conditions led to a total combined production of arachidin-1, arachidin-2, arachidin-3 and arachidin-5 of approximately 750 mg/L in peanut cv. Hull hairy root line 3. Moreover, two hairy root lines from the peanut wild ancestors *A. duranensis* and *A. ipaensis* were developed and characterized in this study. Both of these lines responded to these optimized elicitation conditions with a difference in the prenylated stilbenoid profiles when compared to peanut cv. Hull hairy root line 3. This study demonstrated that orthogonal array design is a feasible approach to boost prenylated stilbenoid production in the peanut hairy root culture system and may lead to successful large-scale production of these bioactive natural products. The two new wild *Arachis* hairy root lines may provide a platform for purification and characterization of other peanut prenylated stilbenoids with potentially different biological activities. 

## Supplementary Materials

The following are available online at https://www.mdpi.com/1420-3049/25/3/509/s1. Figure S1: Comparison of peanut hairy root culture treated with 50 mL and 100 mL elicitation medium. Figure S2: Time course of prenylated stilbenoid yield in the medium of peanut hairy roots after treatment with optimized concentration of elicitors. Figure S3: PCR analysis of *Arachis ipaensis* and *A. duranensis* hairy root lines. Table S1: Elicitation factor and their levels on the orthogonal array design. Table S2: Results and analysis of orthogonal array design L_16_ (4^4^). Table S3: Effects of MeJA and H_2_O_2_ on the production of arachidin-1 and arachidin-3 in peanut hairy root culture co-treated with 18 g/L CD and 1 mM MgCl_2_. Table S4: Time courses of arachidin-1 and arachidin-3 production in peanut hairy root culture co-treated with 3 mM H_2_O_2_, 125 µM MeJA, 18 g/L CD and 1 mM MgCl_2_ in different volume. Table S5: Arachidin-1 and arachidin-3 yield in peanut hairy root culture upon various elicitation conditions. Table S6. Time courses of stilbenoids production in peanut hairy root culture co-treated with 3 mM H_2_O_2_, 125 µM MeJA, 18 g/L CD and 1 mM MgCl_2._
