# Supplementary material for: Production of Prenylated Stilbenoids in Hairy Root Cultures of Peanut (Arachis hypogaea) and Its Wild Relatives A. ipaensis and A. duranensis via an Optimized Elicitation Procedure
Source: Molecules. 2020 Jan 24;25(3):509. doi: 10.3390/molecules25030509 (PMC7037846; doi:10.3390/molecules25030509)
Supplement: Supplementary file 1 [file molecules-25-00509-s001.pdf]

50 mL 100 mL

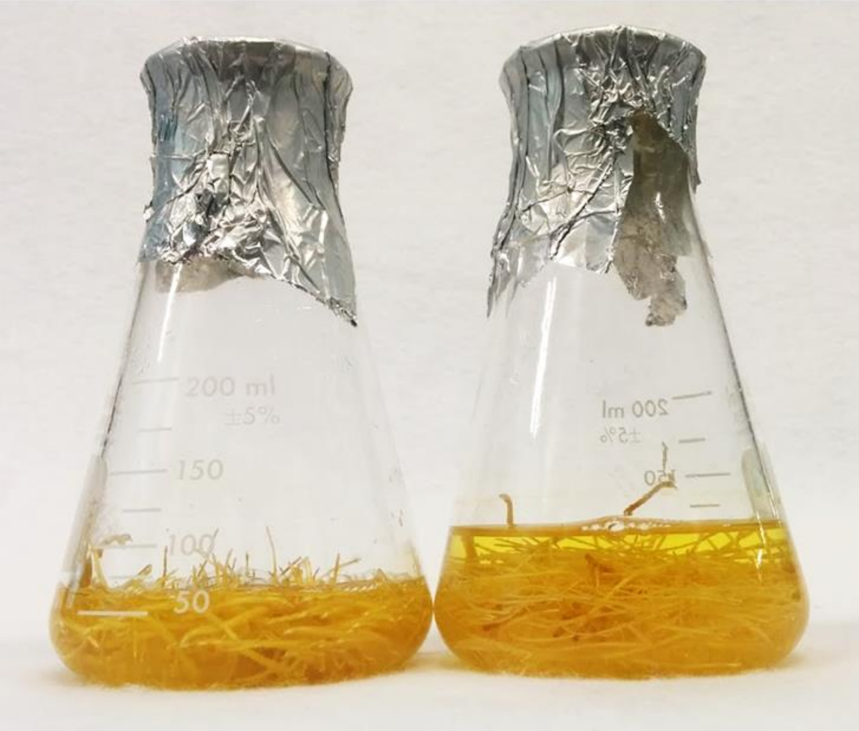

Figure S1. Comparison of peanut hairy root culture treated with 50 mL and 100 mL elicitation medium. Nine-day-old peanut hairy root were treated with 125 µM MeJA, 18 g/L CD, 3 mM H_2O_2 and 1 mM $MgCl_2$ in a 50 mL (left) and 100 mL (right) elicitation medium.

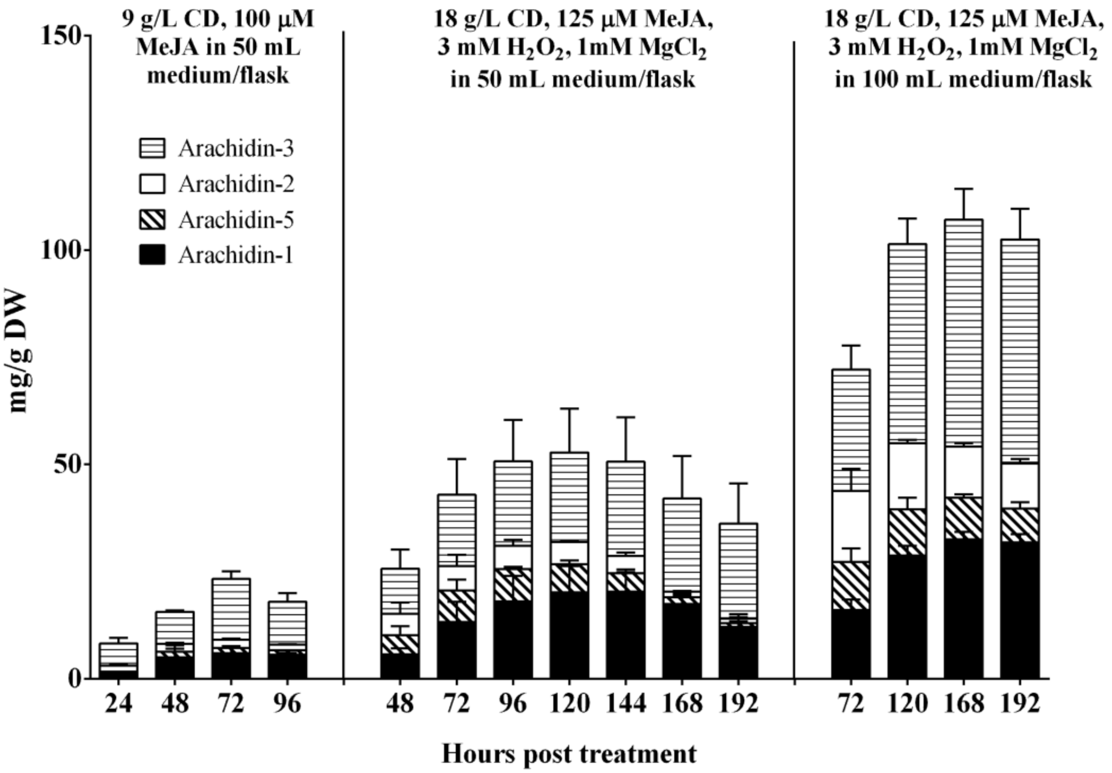

Figure S2. Time course of prenylated stilbenoid yield in the medium of peanut hairy roots after treatment with optimized concentration of elicitors. Prenylated stilbenoids were extracted from medium and quantified by HPLC. Yields are expressed in mg/g of root dry weight**.** Values are the average of three replicates and error bars represent standard deviation.

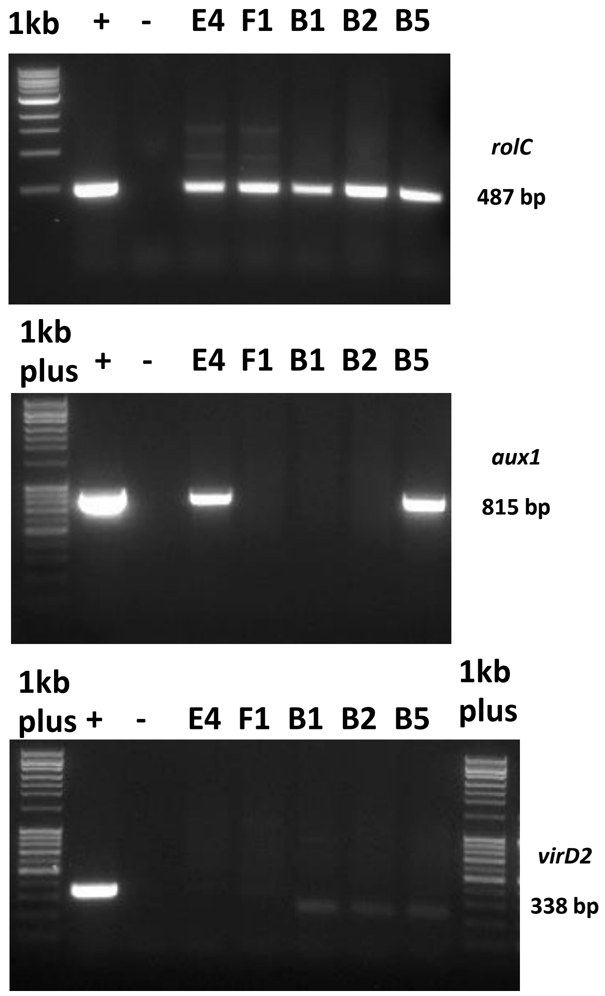

Figure S3. PCR analysis of *Arachis ipaensis* and *A. duranensis* hairy root lines. Genomic DNA was isolated from *A. ipaensis* E4, F1, and *A. duranensis* PI B1, B2, B5. Analyses were performed with primers targeting the*rolC*, *aux1* and *virD2* genes. Plasmid pRi15834 DNA was used as positive control. ddH_2O was used as negative control.

Table S1. Elicitation factor and their levels on the orthogonal array design.

Factors	Levels			
	1	2	3	4
methyl-β-cyclodextrin (CD) (g/L)	4.5	9	18	27
Methyl Jasmonate (MeJA) (μM)	1	10	100	150
H_2O_2 (mM)	6	12	60	120
$MgCl_2$ (mM)	0	1	5	10

Table S2. Results and analysis of orthogonal array design L_{16} (4^4).

Production of arachidin-1 and arachidin-3 in peanut hairy root culture upon the treatment with each combination of elicitors on the orthogonal array are listed on right. Explanation of *k* and *r* values used for orthogonal analysis is described in Materials and Methods.

Run number	CD (g/L)	MeJA (μM)	H_2O_2 (mM)	$MgCL_2$ (mM)	Arachidin-1 (mg/L)		Arachidin-3 (mg/L)	
					48 h	72 h	48 h	72 h
L1	4.5	1	120	5	7.46	17.87	23.45	32.19
L2	4.5	10	60	1	18.45	54.03	57.07	82.03
L3	4.5	100	6	10	109.71	161.20	100.32	93.95
L4	4.5	150	12	0	63.08	110.55	96.60	102.74
L5	9	1	6	0	82.90	140.40	132.94	145.29
L6	9	10	12	10	66.18	124.60	92.36	122.15
L7	9	100	120	1	9.62	23.17	27.21	41.24
L8	9	150	60	5	15.03	46.78	42.91	75.21
L9	18	1	60	10	17.14	47.40	46.20	65.44
L10	18	10	120	0	6.60	19.94	21.41	33.49
L11	18	100	12	5	73.29	162.90	145.59	244.38
L12	18	150	6	1	92.76	226.03	153.51	275.49
L13	27	1	12	1	56.29	119.49	145.83	233.73
L14	27	10	6	5	78.10	180.50	108.71	189.67
L15	27	100	60	0	24.02	59.77	56.34	96.90
L16	27	150	120	10	9.96	28.50	26.00	42.18
k_1^{48}	49.68	40.95	90.87	44.15				
k_2^{48}	43.43	42.33	64.71	44.28				
k_3^{48}	47.45	54.16	18.66	43.47				
k_4^{48}	42.09	45.21	8.41	50.75				
r^{48}	7.59	13.21	82.46	7.28				
k_1^{72}	85.91	81.29	177.03	82.67				
k_2^{72}	83.74	94.77	129.39	105.68				
k_3^{72}	114.07	101.76	52.00	102.01				
k_4^{72}	97.07	102.97	22.37	90.43				
r^{72}	30.33	21.67	154.66	23.01				
$k*_1^{48}$	69.36	87.11	123.87	76.82				
$k*_2^{48}$	73.85	69.89	120.09	95.91				
$k*_3^{48}$	91.68	82.37	50.63	80.16				
$k*_4^{48}$	84.22	79.75	24.52	66.22				
$r*^{48}$	22.32	17.22	99.35	29.68				
$k*_1^{72}$	77.73	119.16	176.10	94.61				
$k*_2^{72}$	95.97	106.83	175.75	158.12				
$k*_3^{72}$	154.70	119.11	79.89	135.36				
$k*_4^{72}$	140.62	123.91	37.28	80.93				
$r*^{72}$	76.97	17.07	138.82	77.19				

k_n^m = ∑(arachidn-1 yield at elicitor level *n* after *m* hrs treatment)/4;

$k*_n^m$ = ∑(arachidn-3 yield at elicitor level *n* after *m* hrs treatment)/4;

$r^m = k_n^m$ (max)- k_n^m (min);

$r*^m = k*_n^m$ (max)- $k*_n^m$ (min).

Table S3. Effects of MeJA and H_2O_2 on the production of arachidin-1 and arachidin-3 in peanut hairy root culture co-treated with 18 g/L CD and 1 mM $MgCl_2$

Run number	MeJA (μM)	H_2O_2 (mM)	Arachidin-1 (mg/L)		Arachidin-3 (mg/L)	
			48 h	72 h	48 h	72 h
1	100	1.5	110.16	202.24	169.94	295.16
2	100	3	108.57	225.60	127.89	199.35
3	100	6	99.96	224.21	136.73	233.54
4	125	1.5	127.01	216.74	192.61	314.30
5	125	3	107.89	231.75	182.43	300.76
6	125	6	111.95	224.16	142.34	220.13
7	150	1.5	128.06	225.67	193.22	310.94
8	150	3	113.88	219.32	155.72	243.62
9	150	6	95.17	179.79	89.88	129.87

Table S4. Time courses of arachidin-1 and arachidin-3 production in peanut hairy root culture co-treated with 3 mM H_2O_2, 125 μM MeJA, 18 g/L CD and 1 mM $MgCl_2$ in different volume.

Treatment Hours	Arachidin-1 (mg/L)		Arachidin-3 (mg/L)	
	50 mL	100 mL	50 mL	100 mL
48	79.34 ± 20.35	-	147.29 ± 62.52	-
72	185.60 ± 65.92	112.01 ± 17.44	232.83 ± 116.46	198.30 ± 39.29
96	252.46 ± 83.23	-	275.74 ± 134.62	-
120	282.65 ± 85.06	201.27 ± 15.91	292.32 ± 143.83	325.11 ± 42.11
144	284.84 ± 63.19	-	307.70 ± 145.16	-
168	244.69 ± 36.85	227.39 ± 12.75	304.39 ± 138.55	370.59 ± 50.37
192	169.66 ± 41.15	222.83 ± 13.67	310.57 ± 131.33	365.85 ± 50.23

-: No data available.

Table S5. Arachidin-1 and arachidin-3 yield in peanut hairy root culture upon various elicitation conditions.

Prenylated stilbenoids were induced by various elicitor(s) and elicitation medium volumes. The yield of arachidin-1 or arachidin-3 secreted into the culture medium was quantified by HPLC and normalized in mg/g of root dry weight.

Treatments	Elicitation volume	Arachidin-1 (mg/g DW)			Arachidin-3 (mg/g DW)		
		48 h	72 h	168 h	48 h	72 h	168 h
100 µM MeJA	50 mL	nd*	nd	-*	0.00032 ± 0.00055	nd	-
100 µM MeJA 9 g/L CD	50 mL	1.48 ± 0.77	2.38 ± 0.63	-	5.74 ± 1.32	10.78 ± 2.12	-
125 µM MeJA, 18 g/L CD 3 mM H_2O_2 and 1 mM $MgCl_2$	50 mL	5.67 ± 1.46	13.26 ± 4.71	17.47 ± 2.63	10.51 ± 4.47	16.63 ± 8.31	21.74 ± 9.90
125 µM MeJA, 18 g/L CD 3 mM H_2O_2 and 1 mM $MgCl_2$	100 mL	-	16.0 ± 2.49	32.49 ± 1.83	-	28.33 ± 5.61	52.94 ± 7.20

nd: Not detected; -: Not available

Table S6. Time courses of stilbenoid production in peanut hairy root culture co-treated with 3 mM H_2O_2, 125 µM MeJA, 18 g/L CD and 1 mM $MgCl_2$

Treatment Hours	*Arachis hypogaea* Hull 3A (mg/L)				
	Resveratrol	Arachidin-5	Arachidin-1	Arachidin-2	Arachidin-3
48	54.52 ± 6.18	30.57 ± 2.72	113.79 ± 4.76	65.32 ± 11.63	88.30 ± 11.48
96	49.43 ± 5.48	41.38 ± 11.79	267.65 ± 22.63	78.59 ± 12.58	141.03 ± 34.68
144	38.13 ± 4.84	32.48 ± 11.04	298.54 ± 41.98	57.21 ± 6.33	155.29 ± 42.82
192	27.18 ± 2.40	22.02 ± 6.61	300.35 ± 36.40	44.28 ± 2.22	178.04 ± 31.15
Treatment Hours	*Arachis ipaensis* E4 (mg/L)				
	Resveratrol	Arachidin-5	Arachidin-1	Arachidin-2	Arachidin-3
48	7.94 ± 3.43	1.62 ± 1.12	36.47 ± 10.56	11.39 ± 7.99	46.70 ± 12.92
96	15.30 ± 3.07	2.66 ± 0.67	65.66 ± 15.02	19.59 ± 6.98	61.37 ± 18.79
144	15.73 ± 2.34	1.99 ± 0.57	63.45 ± 12.61	18.29 ± 3.84	69.32 ± 20.78
192	13.43 ± 3.00	1.39 ± 0.58	55.61 ± 11.86	15.37 ± 3.13	64.07 ± 15.77
Treatment Hours	*Arachis duranensis* B5 (mg/L)				
	Resveratrol	Arachidin-5	Arachidin-1	Arachidin-2	Arachidin-3
48	6.12 ± 0.11	n.d.*	1.57 ± 0.36	7.41 ± 1.40	10.54 ± 1.12
96	8.75 ± 0.09	0.21 ± 0.14	5.93 ± 0.81	11.65 ± 1.64	13.47 ± 1.28
144	9.19 ± 0.18	1.07 ± 0.28	11.17 ± 0.61	14.83 ± 2.10	17.06 ± 1.94
192	8.41 ± 0.22	2.93 ± 0.69	17.93 ± 0.26	17.52 ± 2.30	20.51 ± 3.05

*: Not detected
